# A case of Aspergillus empyema successfully treated by a single-stage cavernostomy and a covering procedure using both fat pad and muscle flap

**DOI:** 10.1186/s44215-023-00109-3

**Published:** 2023-10-26

**Authors:** Yue Cong, Masaaki Nagano, Masaaki Sato

**Affiliations:** Department of Thoracic Surgery, The University of Tokyo, 7-3-1, Hongo, Bunkyo-Ku, Tokyo 113-8655 Japan

**Keywords:** Aspergillus empyema, Pulmonary aspergilloma, Single-stage surgery, Cavernostomy, Fat pad, Latissimus dorsi muscle flap

## Abstract

**Background:**

Aspergillus empyema due to rupture of a pulmonary cavity including an aspergilloma is a serious condition especially in immunocompromised patients with various co-morbidities. Open window thoracotomy is usually performed to control infection, followed by secondary myoplasty. However, such a two-stage strategy requires long treatment period and accompanies the invasiveness of multiple operations. On the other hand, single-stage surgery is minimally invasive, and patients’ activities of daily living are less impaired. We present a single-stage surgery consisting of cavernostomy and a covering procedure using both fat pad and muscle flap.

**Case presentation:**

A 28-year-old man taking 20 mg of prednisone for rheumatoid arthritis presented with right-sided chest pain. A chest computed tomography and thoracoscopy showed a perforated pulmonary cavity including an aspergilloma in the right apex of the lung. Antifungal medication was started, but the thoracic and pulmonary cavities persisted. Therefore, surgical intervention was indicated, and considering the patient’s general condition and anticipated length of treatment period, we decided to conduct a single-stage operation. A cavernostomy was performed on the ruptured cavity. To reliably close the air leak and to occlude the air space to prevent recurrence of infection, a subcutaneous free fat pad was harvested and filled into the cavity. A pedicled latissimus dorsi muscle flap was further introduced into the thoracic space to cover the fat pad. There was no postoperative air leak and the patient was discharged on postoperative day 20 with no adverse events and no limitation of movement in the arm. A series of post-operative CT showed that the pedicled latissimus dorsi muscle flap and free fat pad gradually shrank with lung re-expansion, but they were still present and filled the thoracic and pulmonary cavities 10 months after surgery.

**Conclusion:**

A single-stage surgery consisting of cavernostomy and a covering procedure using both a fat pad and muscle flap was effective in sealing air leaks, filling the air space, and preventing recurrence of infection. The fat pad and muscle flap appear to have worked in a complementary way.

## Background

Aspergillus empyema due to rupture of a pulmonary cavity including an aspergilloma is a rare but serious condition, especially in immunocompromised patients [1–3]. In addition to antifungal medications, surgical treatment is mandatory [4, 5]. Open window thoracotomy (OWT) is usually performed to control infection, followed by second-stage myoplasty to close the open wound [1–5]. However, such a two-stage surgery requires a long treatment period and accompanies the invasiveness of multiple operations. When performing surgical treatment of aspergillus empyema in immunocompromised patients with various co-morbidities, surgeons must consider not only controlling infection to prevent the recurrence of the disease but also the effect of surgical intervention on the patient’s activities of daily living (ADL).

A single-stage surgery consisting of simultaneous cavernostomy and myoplasty has been reported to be minimally invasive and less disabling for patients' ADLs. However, it has also been reported to result in postoperative recurrence of infection [4, 5]. One of the common causes of failure of surgical treatment, especially in single-stage surgery, is residual dead space around the pleural fistula due to inadequate air leak control [4–8]. Several methods to control air leakage have been reported, including bronchial occlusion [6], and muscle flap coverage in combination with drainage [7], but no reliable method has been established yet.

Here we present a case of Aspergillus empyema due to rupture of a pulmonary cavity including an aspergilloma, which was successfully treated by a single-stage surgery consisting of cavernostomy with closure of the air leak and combined fat-pad and muscle-flap coverage to seal air leak and fill the air space.

## Case presentation

**Fig. 1:**
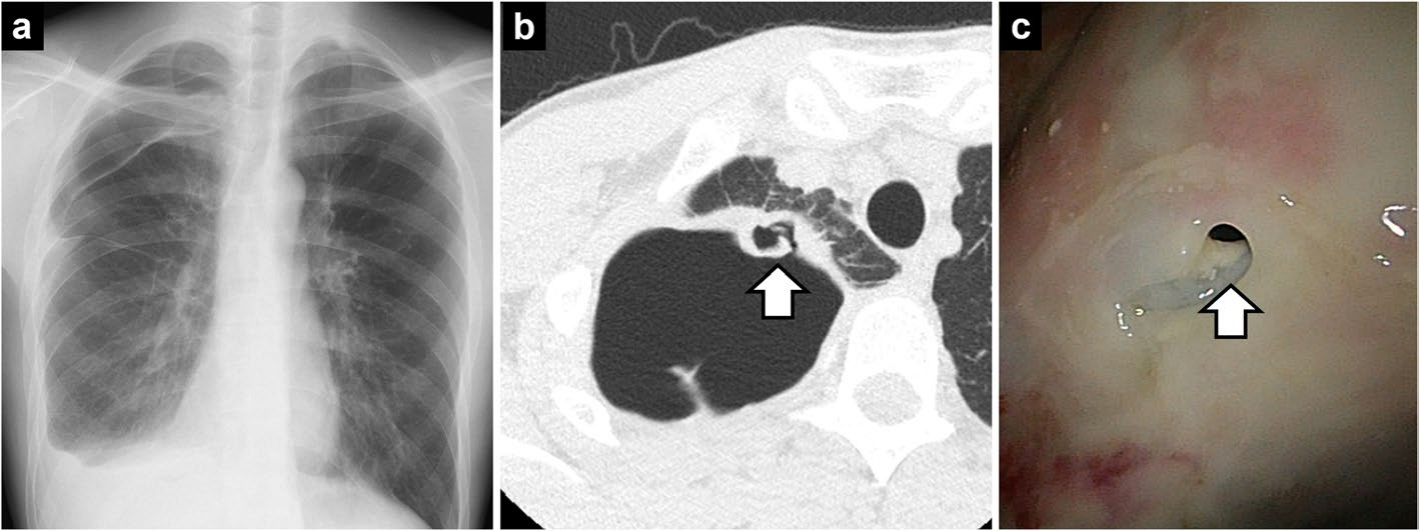
Findings of an aspergillus empyema due to rupture of the cavity of a pulmonary aspergilloma. **A** Chest radiographs showed a right-sided pneumothorax and pleural fluid. **B** Computed tomography showed a perforated lung cavity with a suspected aspergilloma in the right apex of the lung (white arrow). **C** Thoracoscopy revealed a fistula at the apex of the lung (white arrow)

A 28-year-old man was taking 20 mg of prednisone for rheumatoid arthritis. He presented with right-sided chest pain and his chest radiograph showed a right-sided pneumothorax and pleural fluid (Fig. 1a). A chest computed tomography (CT) showed a perforated pulmonary cavity with suspicion of aspergilloma in the right apex of the lung (Fig. 1b). After chest tube drainage, air leakage persisted and lung expansion was inadequate, although the culture of the pleural fluid was negative. Thus, thoracoscopic observation was performed to better examine the thorax and plan an optimal therapeutic strategy. A fistula was found at the apex of the lung (Fig. 1c), and a pleural biopsy demonstrated *Aspergillus fungi*. Antifungal medication (voriconazole) was started, but the thoracic and pulmonary cavities and air leak persisted. Early OWT and subsequent second-stage myoplasty was considered, but there was concern that myoplasty after OWT would increase the amount of muscle to be harvested and have a negative impact on rheumatoid arthritis. In addition, 1 month after antifungal medication and thoracic drainage, the empyema space was decreased and localized to the pleural apex, and there was no pneumonia due to the fistula. Therefore, a single-stage surgery consisting of cavernostomy with closure of the air leak and combined fat-pad and muscle-flap coverage was chosen to control the air leak and fill the remaining dead space.

**Fig. 2:**
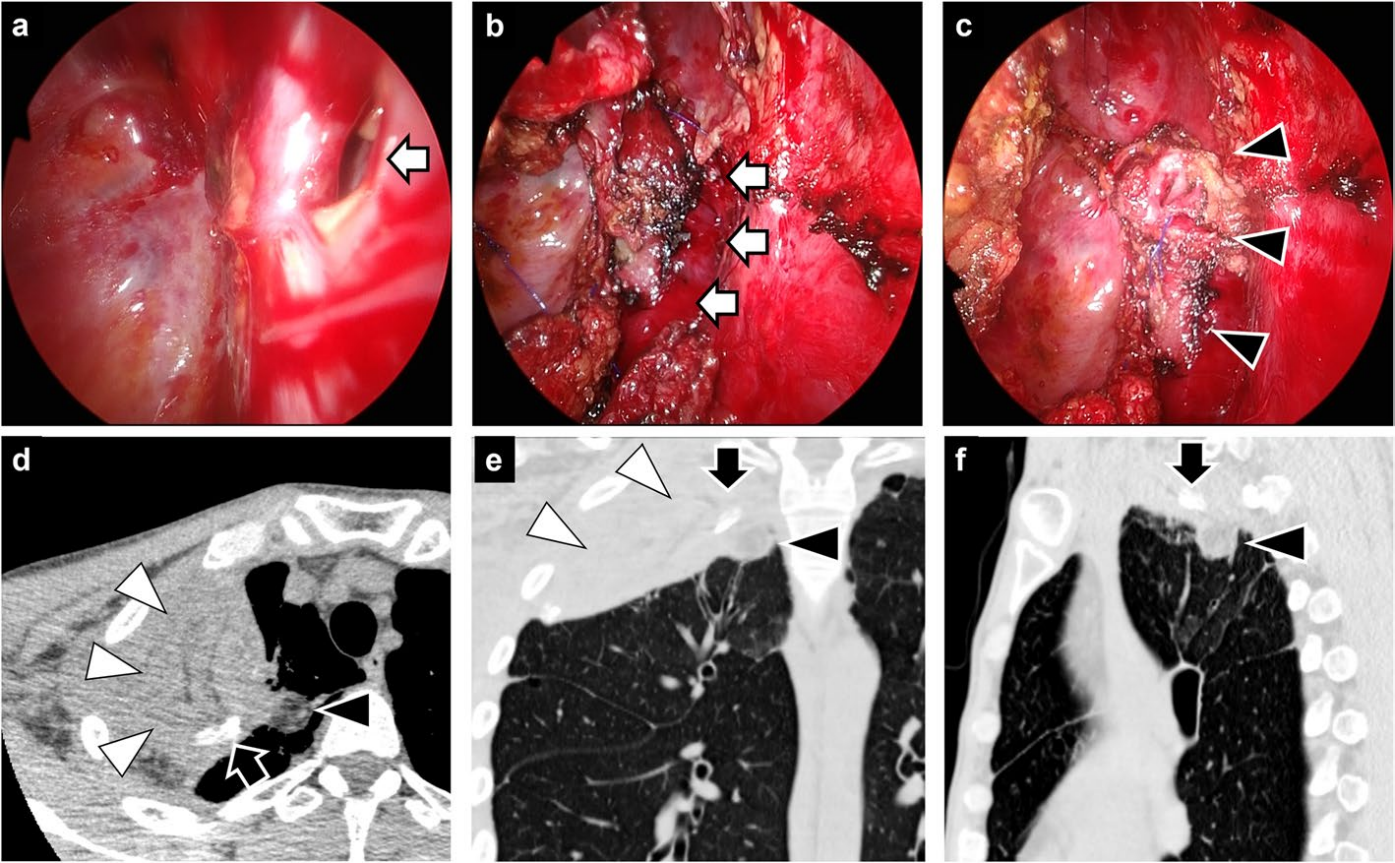
Surgical procedure for single-stage cavernostomy and coverage with combined fat pad and muscle flap. **A** A fistula at the apex of the lung (white arrow). **B** A cavernostomy was performed on the ruptured cavity including aspergilloma (white arrows). **C** A free fat pad was harvested from the skin incision and filled into the ruptured cavity including aspergilloma (black arrowheads). **D**–**F** A pedicled latissimus dorsi muscle flap (white arrowheads) was filled into the thoracic space as a cover for the fat pad (black arrowheads). The black arrow shows a drainage tube placed in the thoracic space

The surgical procedure was as follows: in the left lateral decubitus position, the skin incision was made at the second intercostal space and along the latissimus dorsi muscle, like an inverted λ-shape. A thoracoscope was inserted through the second intercostal space and the pleural cavity was irrigated with saline. A cavernostomy was performed on the ruptured cavity including aspergilloma for debridement and observation of the air leak site (Fig. 2a, b). For reliable closure of the air leak and to fill the ruptured cavity to prevent recurrence of infection, a 3-cm piece of axillary free fat pad was harvested from the skin incision and filled into the ruptured cavity (Fig. 2c). The air leak point was sutured, and the fat pad was fixed by the suture line. Because the volume of the latissimus dorsi muscle flap assessed by CT [9] was sufficient to fill the thoracic cavity, a pedicled latissimus dorsi muscle flap was prepared for coverage. The lateral second intercostal muscle was resected to allow the muscle flap to be filled from the second intercostal space. The muscle flap was filled into the thoracic space and sutured in the cavity wall and lung as a coverage for the fat pad (Fig. 2d, e, f). Because the dead space was fully filled with the fad pad and the muscle, resection of the ribs was avoided, and the thoracic cage was preserved. Two drainage tubes were placed near the ruptured aspergilloma cavity and under the skin from which the muscle flap was taken. The operation time was 108 min, with less than 50 mL of blood loss.

**Fig. 3:**
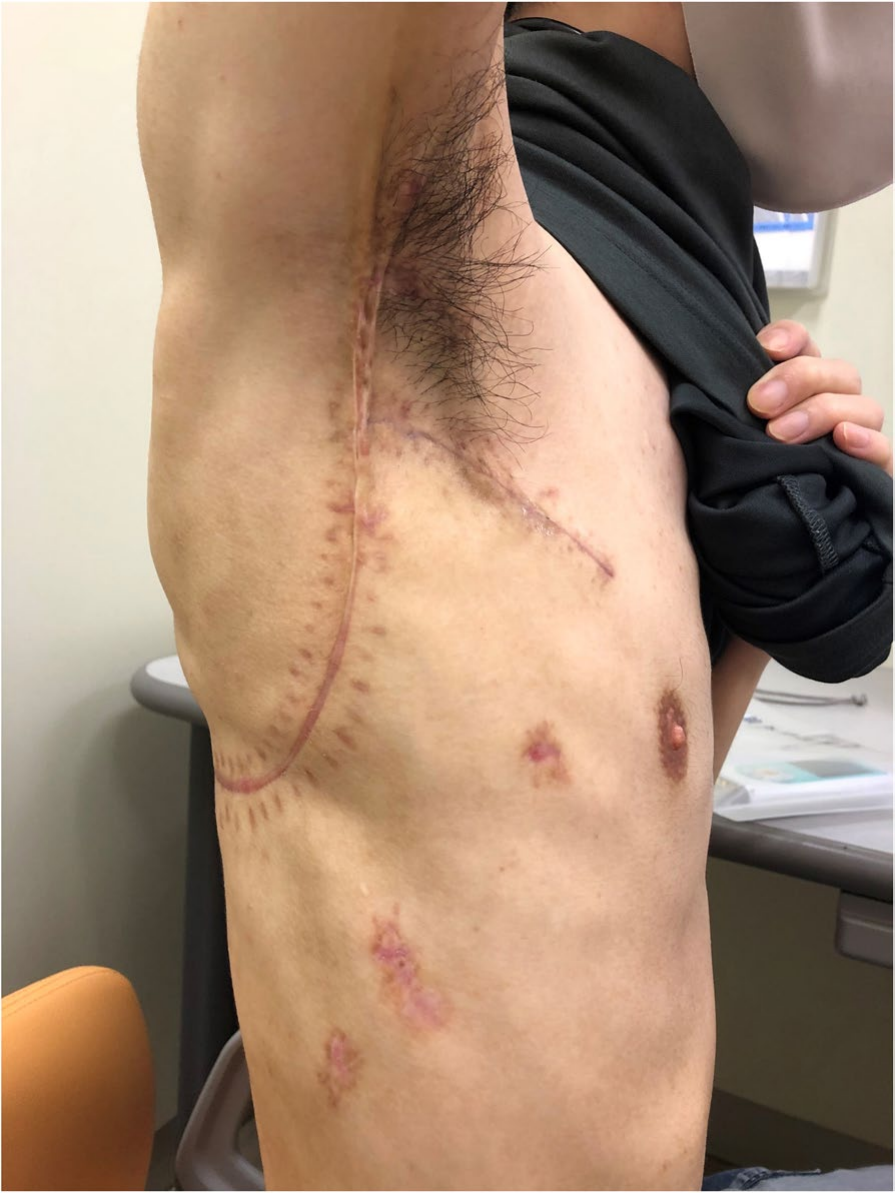
The wound 3 months after surgery. The skin incision resembles an inverted λ-shape. No restriction of movement in the right upper arm

**Fig. 4:**
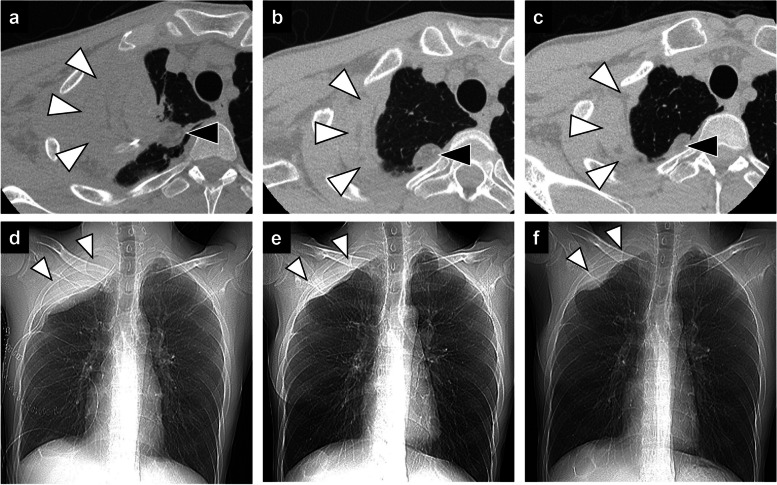
Chest CT follow-up of muscle flap and fat pad. Axial CT (**A**, **B**, **C**) and chest radiographs (**D**, **E**, **F**) at 7 days, 1 month, and 10 months postoperatively. The pedicled latissimus dorsi muscle flap (white arrowheads) and free fat pad (black arrowheads) gradually shrank with lung re-expansion but still filled the thoracic and pulmonary cavities, and the dead space did not recur postoperatively

There was no postoperative air leak and the thoracic drain was removed on postoperative day 7 and the subcutaneous drain on postoperative day 16. Elevation of the patient's upper arm was limited above 90° for 14 days postoperatively to allow the muscle flap to heal. The patient was discharged on postoperative day 20 with no adverse events and no limitation of movement in the right upper arm (Fig. 3). Three months after surgery, the patient started to take additional immunosuppressive medication for rheumatoid arthritis as the infection was under control and there was no recurrence of aspergilloma. Antifungal medication has been continued for a year after surgery to prevent the recurrence of infection. A series of chest CT showed that the pedicled latissimus dorsi muscle flap and free fat pad gradually shrank with lung re-expansion, but they were still present and filled the thoracic and pulmonary cavities even 10 months after surgery (Fig. 4a–c), and thoracoplasty was avoided. There was no recurrence of dead space at 10 months postoperatively.

## Discussion

In the treatment of Aspergillus empyema with air leak, control of air leak and complete filling of the thoracic and pulmonary cavities without leaving dead space are directly related to the success of treatment [4–8]. We successfully treated a case of Aspergillus empyema due to rupture of the cavity of a pulmonary aspergilloma by a single-stage surgery, including cavernostomy, closure of the air leak, and combined fat-pad and muscle-flap coverage.

We used free fat pad to control air leak. Free fat pad has been reported to prevent prolonged post-operative air leak [10–12]. It is easy to harvest and has been reported to persist for several months postoperatively, even without a pedicle [12, 13]. It is important for muscle coverage to reliably control the air leak before filling the muscle flap [4–8], but once the large muscle flap is introduced, the whole pleural cavity is hardly visible, and it is particularly difficult to examine whether the ruptured pulmonary cavity is completely filled. Therefore, we first fixed the free fat pad like a plug at the deepest part of the cavity and the air leak site, where it is most difficult to suture a large muscle flap, and then further covered the fat pad with a muscle flap. The fat pad and muscle flap can work in a complementary way.

For myoplasty, it is important to avoid creating additional dead space during surgery [7, 8]. Although OWT followed by second-stage myoplasty is often performed [6–8], OWT requires rib resection and the subsequent myoplasty needs a larger mass of muscle flap to fill the dead space due to lung deflation [5]. This may critically affect the patient’s ADL, especially arm motion in rheumatoid arthritis patients. Therefore, we tried to avoid thoracoplasty, to minimize the dead space and reduce the surgical invasiveness of myoplasty by single-stage operation and insertion of a muscle flap from the intercostal space without rib resection.

This method, the usage of subcutaneous fat pad and muscle flap for single-stage cavernostomy and combined fat-pad and muscle-flap coverage, has not been reported previously. The treatment strategy was the classic combination of local control of the infection without leaving dead space, to which the fat pad was added to complement the strategy. The indication to adapt this method depends on this strategy. If the drainage is inadequate and pneumonia develops an early OWT must be performed. Also, if the empyema cavity cannot be localized or if the infection continues to spread, this method is difficult to apply. Therefore, as a preliminary step to this method, it is important, to use a thoracoscope if necessary, to confirm the fistula, place the chest tube in the correct position, diagnose the infection, and start antifungal therapy as early as possible. This method would be useful in the surgical treatment of Aspergillus empyema, but further observation is needed to determine the effectiveness of this method because there is no guarantee that the adipose tissue in the infected lesion would function stably in the long run. Furthermore, it is recommended to continue antifungal medication at least for 1 year after surgery to prevent recurrence [14], as recurrence of aspergilloma was reported after a long postoperative period [5]. However, we believe that this combined fat pad and muscle flap covering procedure could be effective and less invasive, especially for immunocompromised patients with various co-morbidities.

## Data Availability

Case report data and the patient’s consent form are available from the corresponding author on reasonable request.

## Abbreviations

ADL: Activities of daily living
CT: Computed tomography
OWT: Open window thoracotomy
